# Fallopian Tube Prolapse after Hysterectomy: A Systematic Review

**DOI:** 10.1371/journal.pone.0076543

**Published:** 2013-10-07

**Authors:** Lobna Ouldamer, Agnès Caille, Gilles Body

**Affiliations:** 1 Department of Gynecology, University Hospital of Tours, Tours, France; 2 INSERM, unit 1069, Tours, France; 3 INSERM, CIC 202, Tours, France; 4 François Rabelais University, Tours, France; Baylor College of Medicine, United States of America

## Abstract

**Background:**

Prolapse of the fallopian tube into the vaginal vault is a rarely reported complication that may occur after hysterectomy. Clinicians can miss the diagnosis of this disregarded complication when dealing with post-hysterectomy vaginal bleeding.

**Objectives:**

We performed a systematic review in order to describe the clinical presentation, therapeutic management and outcome of fallopian tube prolapse occurring after hysterectomy.

**Search Strategy:**

A systematic search of MEDLINE and EMBASE references from January 1980 to December 2010 was performed. We included articles that reported cases of fallopian tube prolapse after hysterectomy. Data from eligible studies were independently extracted onto standardized forms by two reviewers.

**Results:**

Twenty-eight articles including 51 cases of fallopian tube prolapse after hysterectomy were included in this systematic review. Clinical presentations included abdominal pain, dyspareunia, post- coital bleeding, and/or vaginal discharge. Two cases were asymptomatic and diagnosed at routine checkup. The surgical management reported comprised partial or total salpingectomy, with vaginal repair in some cases combined with oophorectomy using different approaches (vaginal approach, combined vaginal-laparoscopic approach, laparoscopic approach, or laparotomy). Six patients were initially treated by silver nitrate application without success.

**Conclusions:**

This systematic review provided a precise summary of the clinical characteristics and treatment of patients presenting with fallopian tube prolapse following hysterectomy published in the past 30 years. We anticipate that these results will help inform current investigations and treatment.

## Introduction

Hysterectomy is the most frequently performed major gynecological surgical procedure [1]. Prolapse of the fallopian tube into the vaginal vault is a rare complication that may occur after hysterectomy, whatever the surgical approach. Any portion of the tube, other than the fimbrial end, may prolapse. The first report of postoperative prolapse of the uterine tube by Pozzi in 1902 described two cases after vaginal hysterectomy [2]. Although the incidence is difficult to estimate, Fan et al suggested than it complicates about 0.1% of hysterectomies [3].

The available literature regarding this complication comprises case reports or case series including few patients.

The aim of our study was to identify, assess and review all relevant series and case reports of fallopian tube prolapse published in the past 30 years. Our specific objectives were to describe i) Individual and clinical characteristics, and ii) the range of therapeutic management and outcomes of patients presenting with fallopian tube prolapse after hysterectomy.

## Methods

### Search Strategy

We systematically searched MEDLINE and EMBASE for relevant English and French language articles. The search terms included “fallopian tube prolapse”, “prolapsed fallopian tube”, “fallopian tube herniation”, and “salpingo-vaginal fistula”. We restricted our search to the period from January 1980 to December 2010 in an attempt to minimize potential bias associated with development of surgical techniques. References were screened for duplicate publications (i.e the same case described in more than one article), and only the most detailed article was included in this analysis. Titles and abstracts of the references retrieved were read by one of the authors (LO). The full texts of the potentially relevant reports were then assessed for eligibility. In addition, the reference lists and the PubMed ‘related articles’ feature were also assessed.

### Inclusion criteria

Articles were included only if they included individual data on patients and if the fallopian tube prolapse was diagnosed after hysterectomy (total or subtotal).

### Data Extraction

Two reviewers (LO and AC) independently extracted data from each article meeting the inclusion criteria, using a standardized form (see Supplementary material, Appendix S1). The data extracted included: 1) article characteristics (year, country, publication type: case report or case series), 2) patients’ medical histories and information on the hysterectomy (type, preoperative and postoperative morbidity), 3) clinical presentation of the fallopian tube prolapse, 4) intervals between hysterectomy and first symptoms of and treatment for fallopian tube prolapse, 5) complementary investigations for diagnosis, and 6) fallopian tube prolapse management and outcomes. When necessary, discrepancies were resolved by discussion between the two reviewers or by referral to a third reviewer (GB).

### Statistical analysis

SAS software (version 9.2 of SAS system for windows; SAS Institute Inc, Cary, NC) was used for statistical analysis. Means and standard deviations (or median and 25^th^ and 75^th^ centiles in case of asymmetrical distribution) are reported for continuous variables. Categorical data are reported as numbers and percentages.

## Results

### Study selection

**Figure 1:**
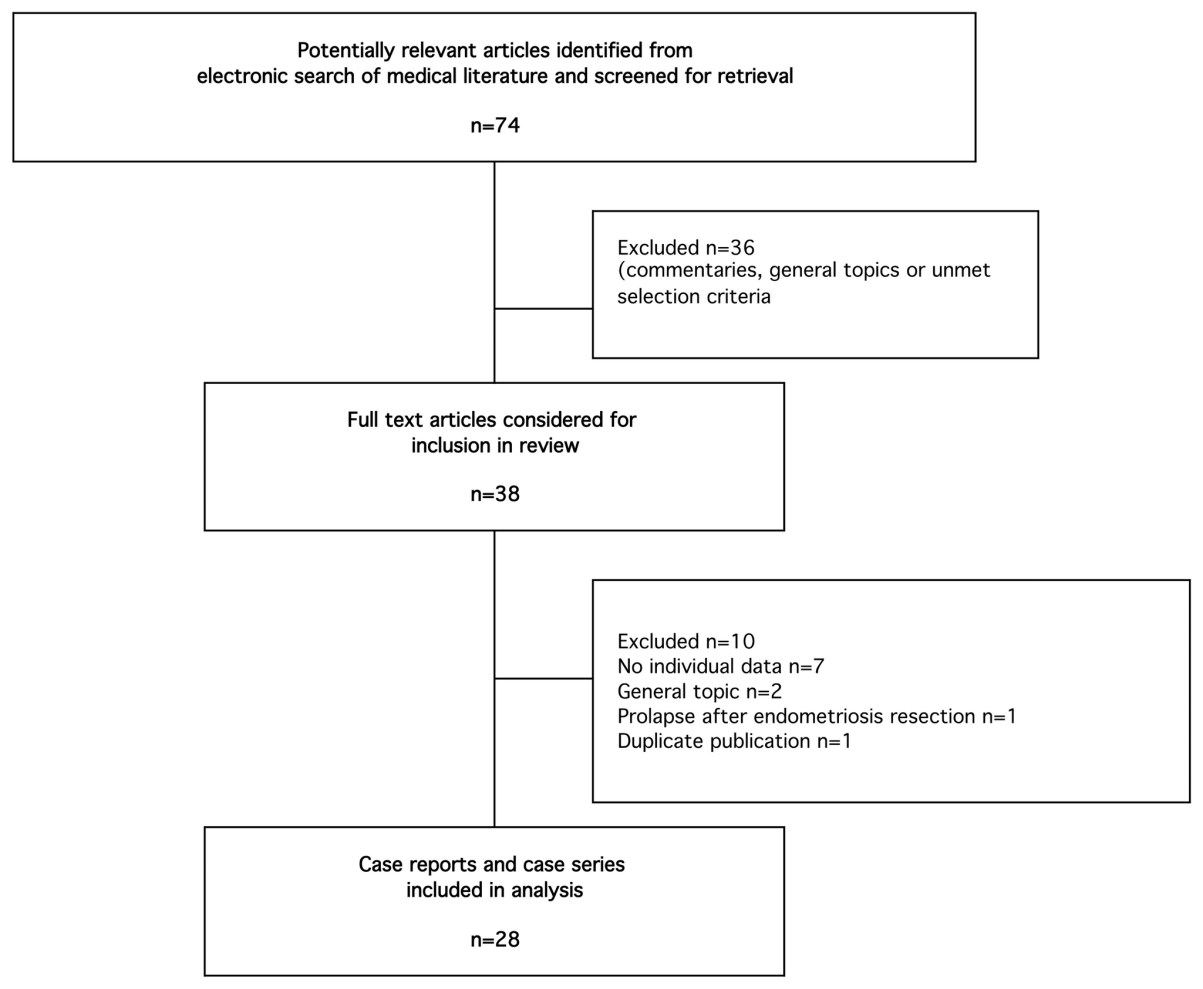
Flow chart of selection process.

Figure 1 summarizes the selection of studies. Of the 74 references identified, only 39 full texts were assessed for eligibility criteria. Of the 39 articles, 11 articles were excluded because there was no individual patient information (n=7), because they concerned general topics (n=2), because it reported fallopian prolapse after laparoscopic resection of endometriosis without hysterectomy (n=1) or because it was a duplicate publication (n=1). Twenty-eight articles were finally included in this systematic review describing 51 patients [4–31].

### Study charateristics

Seventeen of the publications were case reports, ten reported small series of cases (up to seven patients, median number of cases = 3), and one was a letter to the editors. Fifteen of these publications were from Europe, seven from North America, four from Asia, one from Australia, and in one case the country was not specified.

### Patient characteristics

Patient characteristics and history before the diagnosis of fallopian tube prolapse are summarized in Table 1. Mean age of patients presenting with fallopian tube prolapse was 40.0 ± 10.5 years, ranging from 27 to 75 years. Most of the women for whom information was available were parous (n=22/24, 91.7%) with a median of 2.5 deliveries [Interquartile range (IQR): 2-3.5]. The indication for hysterectomy was leiomyomata uteri for 24 patients (47.1%), menometrorrhagia for 14 (27.5%) and other reasons for 13 patients (25.5%) (atypical endometrial hyperplasia n=1, cervical dysplasia with or without invasive component n=3, endometriosis n=1, uterine prolapse n=2, unknown n=6).

**Table 1:** Description of prediagnostic characteristics of patients presenting with fallopian tube prolapse after hysterectomy.

**Characteristics**	**Number of patients with available data**	
Age, years mean (SD)	n=41	40 (10.5)
Premenopausal status	n=41	38 (92.7)
Cause of hysterectomy n (%)	n=51	
Leiomyomata uteri		24 (47.1%)
Menometrorrhagia		14 (27.5%)
Other		13 (25.5%)
Previous surgical procedure n (%)	n=51	
Total abdominal hysterectomy		35 (68.6%)
Subtotal abdominal hysterectomy		1 (2.0%)
Vaginal hysterectomy		9 (17.7%)
Total laparoscopic hysterectomy		4 (7.8%)
Laparoscopic assisted vaginal hysterectomy		2 (3.9%)
Postoperative morbidity n (%)	n=35	
Fever		6 (17.1%)
Cuff hematoma		6 (17.1%)
Other		13 (37.1%)
None		17 (48.7%)
Interval between surgery and symptoms of fallopian tube prolapse,days median (IQR)	n=42	122 (42-365)

IQR, interquartile range, SD, standard deviation

Fallopian tube prolapse was diagnosed after abdominal hysterectomy in 36 patients (70.6%), after vaginal hysterectomy in nine patients (17.7%), after total laparoscopic hysterectomy in four patients (7.8%) and after laparoscopic-assisted vaginal hysterectomy in two patients (3.9%). Hysterectomy was combined with another surgical procedure in six patients (11.8%) (abdominoplasty and abdominal repair n=1, anterior colporrhaphy n=2, left oophorectomy n=2, modified Perreira procedure n=1). The hysterectomy procedure was described for 16 patients, and perioperative complications occurred in five (31.3%) of these 16 patients (abnormal tissue friability n=1, pelvic adhesion n=2, difficulties due to reparative changes n=1, substantial blood loss n=1). Intra-peritoneal vaginal drains were reported for four patients (4/14, 28.6%).

Postoperative morbidity was specifically described for 35 patients, of whom 17 (48.6%) had no complications. Fever was reported for six patients (17.1%) and vaginal cuff hematoma for seven patients (20.0%). Other types of postoperative morbidity were reported for 13 patients: abdominal pain, anemia, low hematocrit values, low hemoglobin concentrations, hematoma, hematoma close to the left ovary, vaginal bleeding, vaginal cuff abscess or cellulitis, wound abscess, wound and pelvic sepsis, encysted empyema, respiratory viral syndrome, micturition problems, subphrenic abscess, urinary tract infection, subacute bowel obstruction, (n=1 for each complication but some patients had more than one complication).

### Clinical presentation of fallopian tube prolapse

The time interval between hysterectomy and first symptoms of fallopian tube prolapse was available for 43 patients: the median interval was 122 days [IQR: 42-365], and the maximum interval was 32 years.

**Figure 2:**
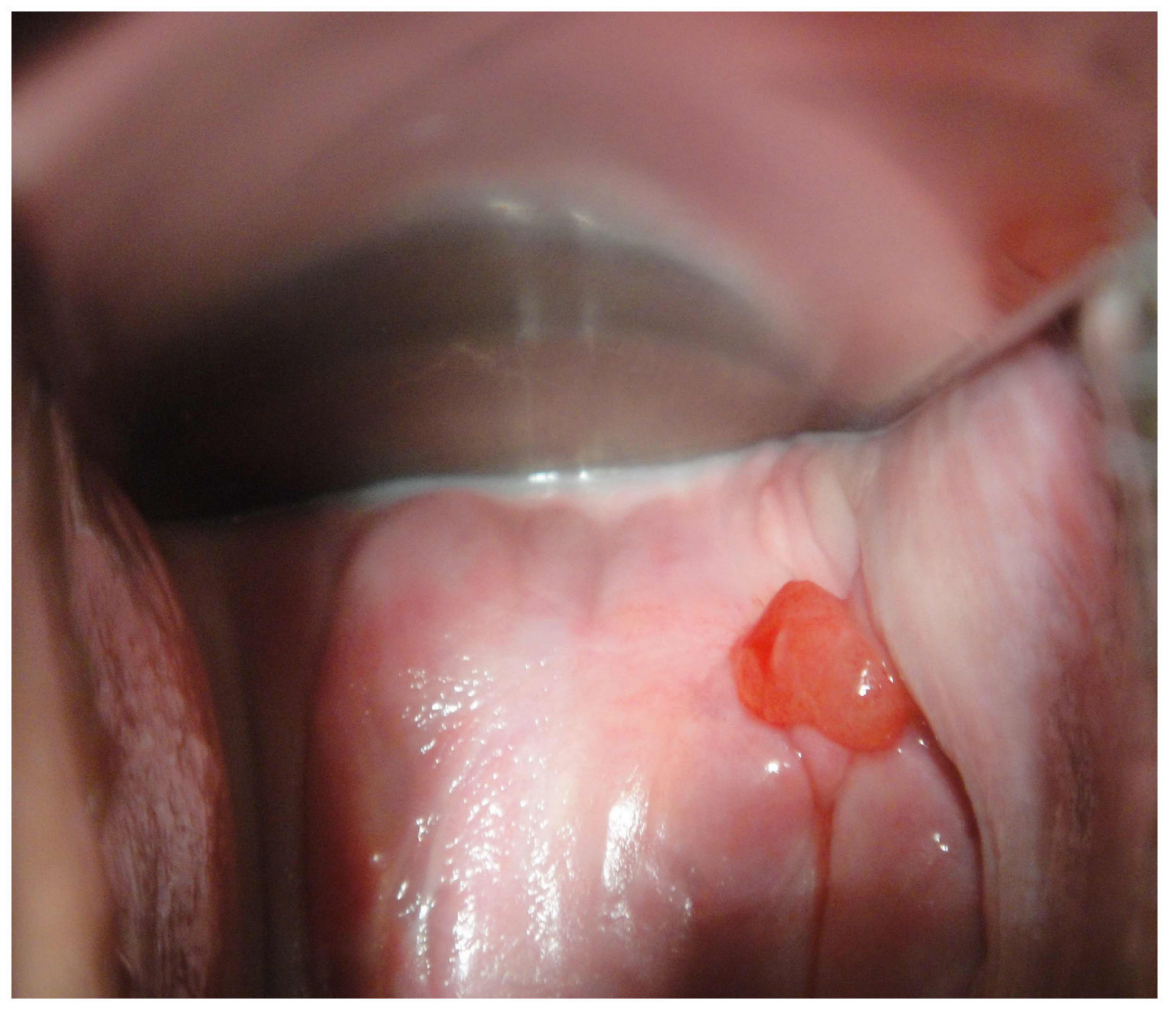
Fallopian tube prolapse mimicking vaginal vault granulation.

The diagnosis suspected before treatment was reported for 39 (76.5%) patients. For 16 patients it was fallopian tube prolapse (41.0%) of which 12 had biopsies, for 14 patients granulation (35.9%) (see Figure 2) of which only one had biopsies, for three patients adenocarcinoma (7.7%) of which two had biopsies, for two patients bladder fistula or incontinence (5.1%), and peritonitis, salpingitis, prolapsed bowel, abcess/hematoma, each for one patient (2.6%).

Of the 50 patients for whom the clinical presentation was specifically described (Table 2), two (4.0%) were asymptomatic and diagnosed at routine checkup. The clinical presentation for the 48 symptomatic patients was mainly characterized by abdominal pain, dyspareunia, post coital bleeding and self-reported vaginal discharge.

**Table 2:** Clinical presentation in patients presenting fallopian tube prolapse after hysterectomy.

**Symptoms**	n* (%)
Abdominal pain	22 (44)
Dyspareunia	17 (34)
Post coital bleeding	12 (24)
Foul-smelling discharge	17 (34)
Bloody discharge	17 (34)
Watery discharge	7 (14)
Other symptoms**	5 (10)
Asymptomatic	2 (4)

* Sum of percentages could be > 100% because one patient may have more than one symptom, the median number of symptoms per patient was 2 [Interquartile range: 1-3].

** Other symptoms were: abdominal distention, fever, mild urgency, nausea/vomiting, dragging sensation (n=1 for each symptom).

### Complementary investigations for diagnosis of fallopian tube prolapse

Biopsies were reported for 17 (33.3%) patients, providing diagnosis of fallopian tube prolapse in 15 cases and suspected adenocarcinoma in two cases that were not confirmed on final pathology. Other investigations were reported for 22 patients; including laboratory blood tests, colposcopy, analysis of vaginal leucorrhea, vaginal swabs, vault smear, cystoscopy, micturating cystogram, intravenous retrograde pyelography, pelvic or vaginal ultrasound, upright abdominal X-ray, sigmoidoscopy, MRI, and laparoscopy.

### Therapeutic management of fallopian tube prolapse and outcome

Information on treatment procedures was available for all patients. The various initial therapeutic approaches are presented in Table 3.

**Table 3:** Initial therapeutic management described for patients presenting fallopian tube prolapse after hysterectomy.

**Therapeutic approach**	**n (%)**
Medical (silver nitrate)	**6 (11.76)**
Vaginal	**28 (54.90)**
Endoloop placement	3
Vaginal excision	10
Partial salpingectomy and vaginal repair	11
Unilateral salpingectomy and vaginal repair	4
Combined vaginal-laparoscopic	**5 (9.80)**
Unilateral salpingectomy and vaginal repair	2
Unilateral salpingo-oophorectomy and vaginal repair	3
Laparoscopic	**6 (11.76)**
Vaginal repair	1
Unilateral salpingectomy and vaginal repair	3
Unilateral salpingo-oophorectomy and vaginal repair	2
Laparotomy	**6 (11.76)**
Unilateral salpingo-oophorectomy	2
Unilateral salpingectomy and vaginal repair	2
Upper vaginectomy with bilateral salpingectomy and pelvic lymphadenectomy	1
Laparotomy without details	1

Information on the interval between hysterectomy and treatment was available for 43 patients, the median interval being 183 days (IQR = 56-365).

Information on the success of the initial treatment was available for 48 patients: 35 (72.9%) had complete recovery after the initial treatment. None of the patients that received silver nitrate recovered (Fisher exact test p<.0001 compared to recovery among patients who received another treatment). None of the patients that received homolateral salpingectomy as a first treatment had recurrence of fallopian tube prolapse.

In one case an ureterovaginal fistula complicated an upper vaginectomy with bilateral salpingectomy and a pelvic lymphadenectomy procedure; this patient underwent ureteroneocystotomy.

**Figure 3:**
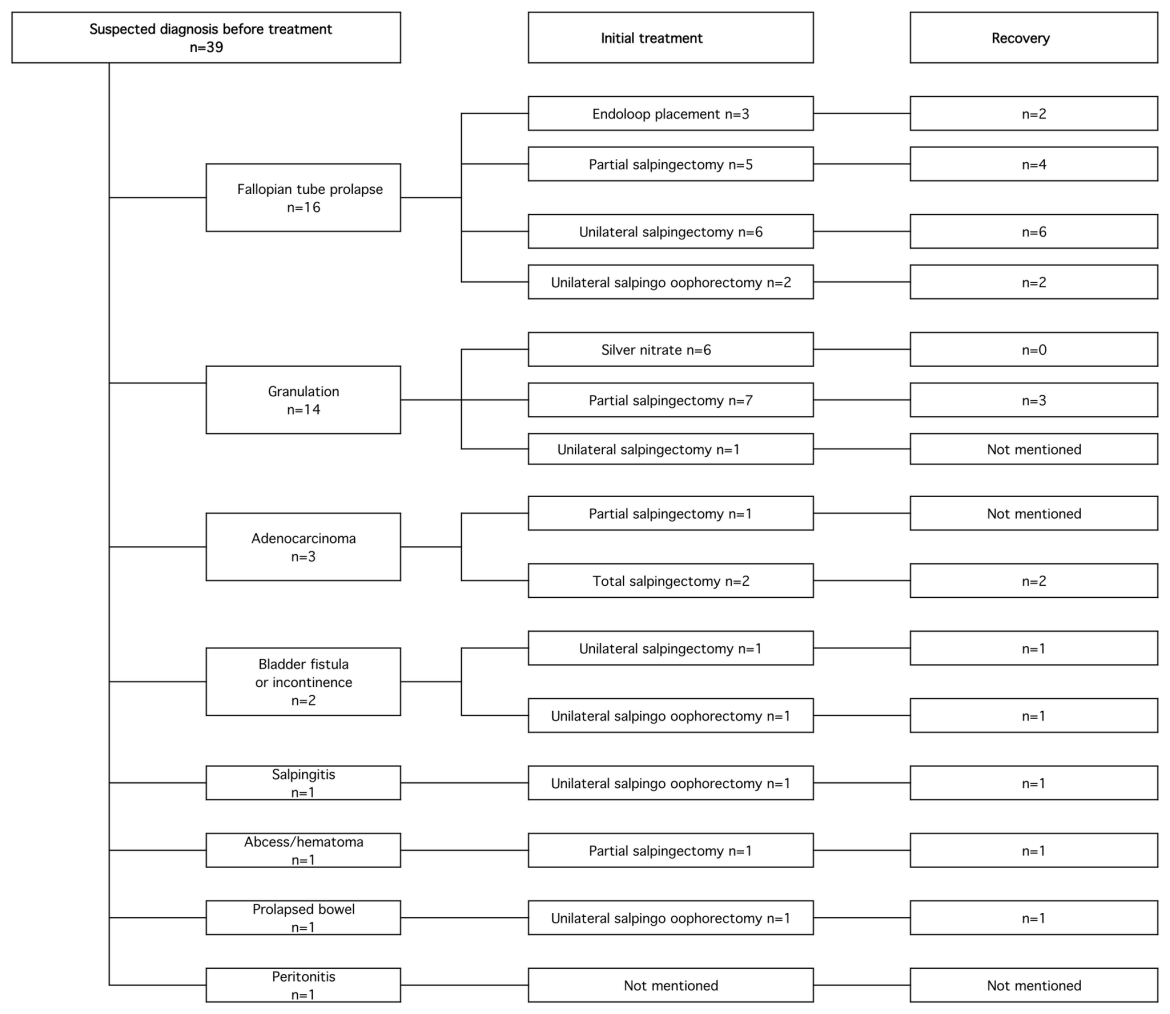
First treatment and outcome with consideration of suspected diagnosis before treatment.

A chart illustrating suspected diagnosis before treatment when available (n=39) with initial treatment and outcome is showed in Figure 3.

Second treatment was needed for 11 patients, in all cases a surgical procedure, with a vaginal approach for five patients (vaginal excision n=2, vaginal partial salpingectomy n=2, endoloop placement n=1), a combined vaginal-laparoscopic approach for two patients (unilateral salpingectomy, n=2), a laparoscopic approach for one patient (unilateral salpingectomy and vaginal repair: n=1), and a laparotomy for three patients (unilateral salpingo-oophorectomy n=2, and unilateral salpingectomy n=1).

Information on the interval between the first and second treatment were available for eight of the 11 patients, the median interval was 84 days (IQR = 37-137).

One patient required a third intervention by laparotomy with removal of the tube stump and suture of the ovary to the round ligament. She finally recovered.

## Discussion

This is the first study to our knowledge that has systematically reviewed the literature on fallopian tube prolapse after hysterectomy. There is insufficient awareness of the possibility of this complication. Indeed, some cases that we retrieved were misdiagnosed as granulation, leading to delayed diagnosis and potentially catastrophic complications (i.e peritonitis). We review here 51 cases of this uncommon complication.

Our review has certain limitations, which are mainly consequences of the relative lack of published data on patients presenting with fallopian tube prolapse. These limited reports may not accurately represent the entire spectrum of the disease and we cannot exclude selection bias due to unreported cases. It is possible that authors tend to write up only unusual cases for publication. This review is further limited by the lack of comprehensive individual information that has not allowed us to describe a full set of variables for all cases.

The mean age of patients who experienced fallopian tube prolapse was 40 years, which is lower than the mean age (46 years) of patients who undergo hysterectomy [32]. Although some of the women were post-menopausal [9,21,26], most of the studies reported patients who were premenopausal [4–8,10–14,16–20,22–24,27–31]. Reasons that may contribute to fallopian tube prolapse are probably multifactorial but the high percentage of premenopausal patients (92.7%) suggest a potential implication of younger age to the occurrence of fallopian herniation. In these patients, early resumption of sexual intercourse before complete healing of the vaginal cuff is suggested to be the main important precipitating event [16,26,30]. Over two thirds of the cases reported in the literature occurred after abdominal hysterectomy. Note that it is widely believed that the laparoscopic approach is associated with an increased risk of vaginal cuff dehiscence [7].

The median interval between hysterectomy and the first symptoms of fallopian tube prolapse was 122 days. Fallopian tube prolapse can occur at any time after hysterectomy and among the cases retrieved was reported as early as during the hysterectomy postoperative period and as late as 32 years after the operation.

Our review revealed reports about two asymptomatic patients with fallopian tube prolapse diagnosed during routine postoperative follow-up. However, most cases were symptomatic, with non-specific and even misleading symptoms. When symptomatic, patients reported profuse vaginal discharge, ranging from clear and watery, mimicking leakage of urine, to bloody; some patients presented with contact bleeding or dyspareunia, and others complained of lower abdominal or pelvic pain. The pain could be reproduced by palpating the protruding prolapsed fallopian tube, but generally the most common clinical finding was red vaginal outpouching (Figure 1). The prolapsed fallopian tube can be mistaken for granulation tissue or a malignant lesion of the vagina, as the prolapsed fallopian tube may lead to distortion or degenerative and atypical changes [9,12,16,21,33]; it can resemble adenocarcinoma and misdiagnosis may result in unnecessary vaginectomy [21,34,35]. Fallopian tube prolapse should therefore be considered in all cases of pelvic or abdominal pain accompanied by vaginal bleeding or discharge after hysterectomy, with or without granulation tissue in the vaginal vault.

The differential diagnosis may include granulation tissue related to surgery, vaginal adenosis, endometriosis, and primary or metastatic adenocarcinoma. Vesicovaginal and ureterovaginal fistulae should also be included [6,10,36].

A diagnosis of formation of vaginal vault granulation should be reconsidered if the tissue observed is resistant to cauterization. Several techniques have been used to diagnose fallopian tube prolapse, and biopsy was the most frequent diagnostic modality in our study (33.3% of published cases); it correctly diagnosed 15 of the 17 cases. A biopsy should be taken from any lesion within the vaginal vault which bleeds easily and persists despite cauterization. Histology allows definitive diagnosis: intact fimbrial structures normally covered with columnar epithelium can be recognized grossly or microscopically, even if there is remodeling of the tube structure with pseudoglandular or polyp-like formations [10]. Careful microscopic analysis reveals the presence of a typical tube epithelium in all cases [35,37]. Nevertheless, failure to detect typical tube epithelium, and background granulation tissue, may lead to misdiagnosis, including histopathologic diagnosis of malignancy. Pankeratin antibodies can be used to detect fallopian tube epithelium immunohistochemically, and this is a valuable approach [10]. Other investigations have been described in the literature to rule out differential diagnosis in fallopian tube prolapse, for example cystoscopy to rule out vesicovaginal fistula.

There is currently no consensus about the ideal method of surgical repair. Of the 51 fallopian tube prolapses that were reported, 11 patients experienced a subsequent herniation that required a second repair, and one patient even required a third intervention. Many factors affect the choice of surgical repair technique (surgeon’s experience, the possibility of performing salpingectomy and of reapproximating the vaginal mucosa appropriately), and treatment should be decided on a case-by-case basis. The choice between abdominal, vaginal, and combined laparoscopic approaches depends on the individual case and circumstances: a vaginal or laparoscopic route, or a combined laparoscopic and vaginal approach, are usually used but the surgical procedure can be complicated by adhesions between the vaginal vault, bowel, bladder and adnexal structures. Total salpingectomy with closure of the vault defect is considered to be the optimal management because partial salpingectomies can result in recurrence of vaginal discharge, and continuing traction on the tubal remnant can lead to persistent pain and dyspareunia [8,18,24,38–40]. Unlike the vaginal approach, the combined laparoscopic-vaginal approach offers the potential benefits of inspection of the entire abdominal cavity and meticulous lavage of the peritoneal cavity and appears to minimize the inherent morbidity associated with laparotomy.

The reasons that may contribute to fallopian tube prolapse are probably multifactorial: for the fallopian tube prolapse to occur after hysterectomy, a communication must develop between the peritoneal cavity and the vagina, and there must be a fallopian tube segment of sufficient length and mobility, that may result from a defective operative technique or difficulties in closing the vaginal cuff. However, certain factors may predispose to tube prolapse, including poor physical condition of the patient (abnormal tissue friability, malnutrition, poorly controlled diabetes mellitus, chronic cough and chronic constipation), postoperative cuff infection or hematoma, pelvic infection, and conditions that impair proper wound healing (chronic steroid use, malignancy and tissue radiation).

Systematic salpingectomies during conservative hysterectomies may be an appropriate approach to prevent fallopian tube prolapse. It would prevent not only fallopian tube prolapse, but also tubal and serous ovarian cancer [41–49]. Clinical trials that directly compare the effects of salpingectomy with fallopian tube preservation on subsequent complications after hysterectomy are needed to support these suggestions. They should also ensure the absence of deleterious effects on ovarian function.

## Supporting Information

Appendix S1 **Extraction form.**
(DOC)

Checklist S1 (DOC)
