# Supplementary material for: Fallopian Tube Prolapse after Hysterectomy: A Systematic Review
Source: PLoS One. 2013 Oct 7;8(10):e76543. doi: 10.1371/journal.pone.0076543 (PMC3792027; doi:10.1371/journal.pone.0076543)
Supplement: Appendix S1 — Extraction form. (DOC) [file pone.0076543.s001.doc]

Data extraction: Fallopian Tube Prolapse after Hysterectomy

## Article characteristics

First author:

Publication year: 

Journal (abbreviation):

Country:

City:

Medical center:

Publication type:  Case report

Case serie

Other (*please specify*):

If case serie, number of cases reported 

Inclusion criteria:  English or French language

Individual data on patient(s)

Hysterectomy

## Patient characteristics

### First patient (youngest)

Patient number:  (Article number then patient number)

Age: 

Parous :  Yes

No

Not mentioned

If Yes, Parity (number of deliveries) : 

Cause of hysterectomy :  Leiomyomata uteri

(one item or more)  Menometrorrhagia

Not mentioned

Other (*please specify*):

**Hysterectomy**

Surgery :  Abdominal hysterectomy (AH)

(one item or more)  Laparoscopic hysterectomy (=cœlioscopic) (TLH)

Laparoscopic assisted vaginal hysterectomy (LAVH)

Vaginal hysterectomy (VH)

Not mentioned

Other (*please specify*):

Complication during the surgical procedure:  Uncomplicated surgical procedure

Complicated surgical procedure

No data on complication during

surgical procedure

If complicated, (*please specify*):

Vaginal “drain” :  Yes

No

Not mentioned

**Postoperative morbidity**

(say No if it’s written that the patient did not have the complication or if patient have at least another event mentioned, say Not mentioned in other case)

Fever:  Yes

No

Not mentioned

Vaginal cuff hematoma:  Yes

No

Not mentioned

Parietal hematoma:  Yes

No

Not mentioned

Salpingitis:  Yes

No

Not mentioned

Other post operative morbidity:  Yes

No

Not mentioned

If yes, (*please specify*):

Complication during postoperative course:  Uncomplicated

Complicated = at least one event

No data on postoperative morbidity (if Not mentioned to previous 6 questions)

**Presenting symptoms**

(say No if it’s written that the patient did not have the symptom or if patient have at least another symptom mentioned, say Not mentioned in other case)

Asymptomatic:  Yes

No

Not mentioned

Abdominal or pelvic pain :  Yes

No

Not mentioned

Dyspareunia :  Yes

No

Not mentioned

Postcoïtal bleeding :  Yes

No

Not mentioned

Foul smelling discharge  Yes

No

Not mentioned

Watery discharge :  Yes

No

Not mentioned

Bloody discharge :  Yes

No

Not mentioned

Other symptom:  Yes

No

Not mentioned

If yes, (*please specify*):

**Investigations**

Biopsy (at any time):  Yes

No

If yes, results of the biopsy:  Fragment of fallopian tube

Granulation tissue

Adenocarcinoma

Not mentioned

Other (*please specify*):

If no biopsy, let this item empty.

Other investigation:  Yes

No

If yes, (*please specify*):

**Delays**

For delays, if not mentioned: NS one month = 30.4375 days one year = 365.25 days

Delay between hysterectomy and first complaints (days): ,

Delay between hysterectomy and first treatment of prolapse (days): ,

Suspected diagnosis before biopsy :  Not mentioned

(complete only if Biopsy = Yes)  Fallopian tube prolapse

Granulation

Adenocarcinoma

Incontinence

Other (*please specify*):

Suspected diagnosis before first treatment :  Not mentioned

Granulation

Fallopian tube prolapse

Adenocarcinoma

Incontinence

Other (*please specify*):

**Treatment of prolapse**

First treatment (after first visit) :

Endoloop placement

Laparoscopy: bilateral salpingectomy and vaginal repair

Laparoscopy: bilateral salpingo oophorectomy and vaginal repair

Laparoscopy: unilateral salpingectomy and vaginal repair

Laparoscopy: unilateral salpingo oophorectomy and vaginal repair

Laparoscopy: vaginal repair

Laparotomy: bilateral salpingectomy and vaginal repair

Laparotomy: bilateral salpingo oophorectomy and vaginal repair

Laparotomy: unilateral salpingectomy and vaginal repair

Laparotomy: unilateral salpingo oophorectomy and vaginal repair

Laparotomy: upper vaginectomy, bilateral salpingectomy and pelvic lympha

Silver nitrate

Vaginal excision

Vaginal bilateral salpingectomy and vaginal repair

Vaginal partial salpingectomy and vaginal repair

Vaginal unilateral salpingectomie and vaginal repair

Vaginal-laparoscopic approach: unilateral salpingectomie and vaginal repair

Vaginal-laparoscopic approach: unilateral salpingo-oophorectomy and vaginal repair

Vaginal-laparoscopic approach: bilateral salpingectomie and vaginal repair

Vaginal-laparoscopic approach: bilateral salpingo-oophorectomy and

No treatment

Not mentioned

Other (please specify):

Recovery after first treatment:  Yes

No

Not mentioned

Follow-up after first treatment if recovery (days): ,

Complication after first treatment:  Yes

No

Not mentioned

If complicated, (*please specify*):

Second treatment:  Yes

No

Not mentioned

Delay between first and second treatment (days) : ,

Second treatment :

Endoloop placement

Laparoscopy: bilateral salpingectomy and vaginal repair

Laparoscopy: bilateral salpingo oophorectomy and vaginal repair

Laparoscopy: unilateral salpingectomy and vaginal repair

Laparoscopy: unilateral salpingo oophorectomy and vaginal repair

Laparoscopy: vaginal repair

Laparotomy: bilateral salpingectomy and vaginal repair

Laparotomy: bilateral salpingo oophorectomy and vaginal repair

Laparotomy: unilateral salpingectomy and vaginal repair

Laparotomy: unilateral salpingo oophorectomy and vaginal repair

Laparotomy: upper vaginectomy, bilateral salpingectomy and pelvic lympha

Silver nitrate

Vaginal excision

Vaginal bilateral salpingectomy and vaginal repair

Vaginal partial salpingectomy and vaginal repair

Vaginal unilateral salpingectomie and vaginal repair

Vaginal-laparoscopic approach: unilateral salpingectomie and vaginal repair

Vaginal-laparoscopic approach: unilateral salpingo-oophorectomy and vaginal repair

Vaginal-laparoscopic approach: bilateral salpingectomie and vaginal repair

Vaginal-laparoscopic approach: bilateral salpingo-oophorectomy and

No treatment

Not mentioned

Other (please specify):

Recovery after second treatment:  Yes

No

Not mentioned

Follow-up after second treatment if recovery (days): ,

Complication after second treatment:  Yes

No

Not mentioned

If complicated, (*please specify*):

Third treatment:  Yes

No

Not mentioned

Delay between second and third treatment (days) : ,

Third treatment :

Endoloop placement

Laparoscopy: bilateral salpingectomy and vaginal repair

Laparoscopy: bilateral salpingo oophorectomy and vaginal repair

Laparoscopy: unilateral salpingectomy and vaginal repair

Laparoscopy: unilateral salpingo oophorectomy and vaginal repair

Laparoscopy: vaginal repair

Laparotomy: bilateral salpingectomy and vaginal repair

Laparotomy: bilateral salpingo oophorectomy and vaginal repair

Laparotomy: unilateral salpingectomy and vaginal repair

Laparotomy: unilateral salpingo oophorectomy and vaginal repair

Laparotomy: upper vaginectomy, bilateral salpingectomy and pelvic lympha

Silver nitrate

Vaginal excision

Vaginal bilateral salpingectomy and vaginal repair

Vaginal partial salpingectomy and vaginal repair

Vaginal unilateral salpingectomie and vaginal repair

Vaginal-laparoscopic approach: unilateral salpingectomie and vaginal repair

Vaginal-laparoscopic approach: unilateral salpingo-oophorectomy and vaginal repair

Vaginal-laparoscopic approach: bilateral salpingectomie and vaginal repair

Vaginal-laparoscopic approach: bilateral salpingo-oophorectomy and

No treatment

Not mentioned

Other (please specify):

Recovery after third treatment:  Yes

No

Not mentioned

Follow-up after third treatment if recovery (days): ,

Complication after third treatment:  Yes

No

Not mentioned

If complicated, (*please specify*):

### Second patient

…..

### Last patient (oldest)

Comments:

Name:
